# *Orthohepevirus* C: An Expanding Species of Emerging Hepatitis E Virus Variants

**DOI:** 10.3390/pathogens9030154

**Published:** 2020-02-25

**Authors:** Bo Wang, Dominik Harms, Xing-Lou Yang, C.-Thomas Bock

**Affiliations:** 1 Department of Biomedical Sciences and Pathobiology, Virginia-Maryland College of Veterinary Medicine, Virginia Polytechnic Institute and State University, Blacksburg, VA 24061, USA; bowang@vt.edu; 2 Department of Infectious Diseases, Division of Viral Gastroenteritis and Hepatitis Pathogens and Enteroviruses, Robert Koch Institute, 13353 Berlin, Germany; HarmsD@rki.de; 3 CAS Key Laboratory of Special Pathogens, Wuhan Institute of Virology, Center for Biosafety Mega-Science, Chinese Academy of Sciences, Wuhan 430071, China; yangxl@wh.iov.cn; 4 Institute of Tropical Medicine, University of Tübingen, 72074 Tübingen, Germany

**Keywords:** *Orthohepevirus* C, hepatitis E virus, genetic variability, molecular evolution, host, zoonosis

## Abstract

Hepatitis E virus (HEV) is an emerging zoonotic pathogen that has received an increasing amount of attention from virologists, clinicians, veterinarians, and epidemiologists over the past decade. The host range and animal reservoirs of HEV are rapidly expanding and a plethora of emerging HEV variants have been recently identified, some of which have the potential for interspecies infection. In this review, the detection of genetically diverse HEV variants, classified into and presumably associated with the species *Orthohepevirus* C, currently comprising HEV genotypes C1 and C2, by either serological or molecular approach is summarized. The distribution, genomic variability, and evolution of *Orthohepevirus* C are analyzed. Moreover, the potential risk of cross-species infection and zoonotic transmission of *Orthohepevirus* C are discussed.

## 1. Introduction

Hepatitis E Virus (HEV) is the causative viral pathogen of hepatitis E, originally considered a self-limiting disease occurring only in developing countries with poor sanitary conditions [1]. Mortality rates in infected pregnant women can reach up to 25% during hepatitis E outbreaks in these countries [2]. In the late 1990s, hepatitis E was recognized to be a zoonotic disease and swine was found to be the natural host of HEV in the USA. Soon afterwards, a large number of autochthonous cases were reported in different Europe countries associated with the consumption of undercooked meat [3]. Hepatitis E poses a significant health risk for immunocompromised individuals, such as solid organ transplant recipients and patients with human immunodeficiency virus infection, in whom chronic infections can occur. Ribavirin is commonly administered as an off-label treatment for chronic HEV infection, but significant side-effects often limit its use [4]. Furthermore, HEV may also cause extrahepatic manifestations, including renal and neurological injuries, pancreatitis, cryoglobulinemia, and hematological disorders [5]. In light of this, hepatitis E is being increasingly recognized as a serious public health burden worldwide.

HEV was first isolated in 1983 and the viral genome was subsequently cloned and sequenced [6,7]. HEV is approximately 27–34 nm in diameter. Viral genomes are 6.4–7.2 kb long capped positive-sense single-stranded RNA containing three open readings frames (ORF1, ORF2, and ORF3), 5′- and 3′- untranslated regions, a 7-methylguanylate cap at the 5′ end and a polyadenine tract at the 3′-end. ORF1 encodes a non-structural polyprotein containing a methyltransferase, papain-like cysteine protease, macro domain, RNA helicase, and RNA-dependent RNA polymerase. ORF2 encodes the capsid protein. ORF3 overlaps partially with ORF2 and encodes a multifunctional phosphoprotein associated with viral egress [8]. Recent studies have reported that HEV particles are non-enveloped and infectious in bile and feces, while quasi-enveloped and less infectious/non-infectious in sera [9].

HEV differs from all other human hepatitis viruses in that it is closely related to non-human viruses [10]. HEV is the prototype of the family *Hepeviridae*, which includes the genera *Orthohepevirus* and *Piscihepevirus*. The genus *Orthohepevirus* is further divided into four species *Orthohepevirus* A to D. *Orthohepevirus* A contains HEV variants isolated from human, pig, wild boar, deer, mongoose, rabbit, and camel; *Orthohepevirus* B from chicken; *Orthohepevirus* C from rat, greater bandicoot, Asian musk shrew, ferret, and mink; and *Orthohepevirus* D from bat. The genus *Piscihepevirus* occurs in cutthroat trout and possibly other fish species [11]. Based on the host range of viruses and phylogeny of viral genomic sequences, proposals are also made for the designation of HEV genotypes and subgenotypes [12]. Nevertheless, numerous novel HEV strains remain unclassified due to the large degree of divergence or the lack of complete genomic sequences.

The species *Orthohepevirus* C is separated into two genotypes: HEV-C1 and HEV-C2. HEV-C1 contains variants derived from hosts in the orders Rodentia and Soricomorpha, and HEV-C2 from the order Carnivora [8,11]. By means of the application and development of new molecular techniques such as metagenomic sequencing, a growing number of distinct HEV variants have been discovered in a variety of animal species globally and the species *Orthohepevirus* C has been rapidly extended in the last years [13]. Although the zoonotic potential of *Orthohepevirus C* is still under debate, two recent clinical cases describing persistent hepatitis in a liver transplant patient in Hong Kong and severe acute hepatitis in an immunocompetent patient in Canada, respectively, have been attributed to infection with rat HEV [14,15]. Furthermore, seven additional rat HEV infections have been confirmed lately in Hong Kong [16]. In this review, we aim to compile and discuss the recent discoveries of HEV variants within the species *Orthohepevirus* C and their potential risk of cross-species infection and zoonotic transmission to humans.

## 2. Detection and Distribution of *Orthohepevirus* C

**Figure 1:**
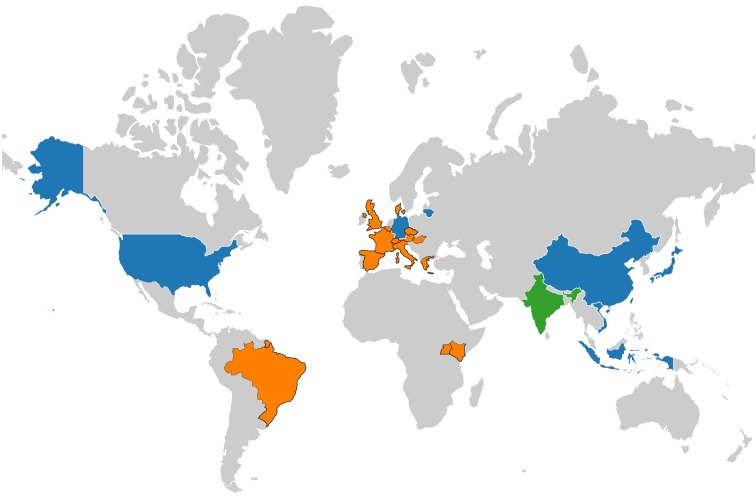
Worldwide detection and distribution of *Orthohepevirus* C. Countries with both *Orthohepevirus* C RNA and antibodies detection are in blue; countries with only antibodies detection are in green; countries with only RNA detection are in orange. World map was created by using a free and open source geographic information system Quantum Geographic Information System version 3.10 (http://qgis.osgeo.org) and free vector and raster map data from Natural Earth (http://www.naturalearthdata.com).

The first study concerning the prevalence of anti-HEV antibodies in wild rats was conducted in the USA in 1999 [17]. In 2000, another study from the USA reported on the HEV seroprevalence among 13 rodent species, providing evidence for widespread infection of *Orthohepevirus* C in rodents and suggesting these animals might be the natural reservoirs for this species of HEV [18]. Subsequently, *Orthohepevirus* C RNA was detected in feces of Norway rats in Germany in 2010 using a nested broad-spectrum reverse transcription polymerase chain reaction (RT-PCR) and the complete genomes were sequenced and found to be highly divergent from human HEV strains [19,20]. Thus far, anti-HEV or anti-rat HEV antibodies have been detected in 23 animal species, including 12 and 10 species within the families *Muridae* and *Cricetidae* of the order Rodentia, respectively, and one species within the family *Soricidae* of the order Soricomorpha, from eight countries (the USA, Japan, Germany, China, Vietnam, Lithuania, Indonesia, and India). Additionally, *Orthohepevirus* C sequences have been detected in 22 countries (the USA, Germany, France, Denmark, China, Lithuania, Hungary, Austria, Switzerland, Italy, Spain, Greece, Belgium, Czech Republic, England, Indonesia, Vietnam, Uganda, the Netherlands, Japan, Brazil, and Kenya) and 25 animal species, containing eight species within the family *Muridae* and 10 species within the family *Cricetidae* of the order Rodentia, one species within the family *Ursidae* and two species within the family *Mustelidae* of the order Carnivore, two species within the family *Falconidae* of the order Falconiformes, one species within the family *Soricidae* of the order Soricomorpha, and one species within family *Hominidae* of the order Primates. Worldwide detection and distribution of *Orthohepevirus* C is displayed in Figure 1. Although HEV serology and nucleic acid detection remains negative in several of these animal species, this may be linked to limitations of current detection assays, such as poor specificity and low sensitivity.

### 2.1. Seroprevalence of Anti-HEV Antibodies

To date, 11 seroprevalence studies have been carried out on *Rattus norvegicus* (Brown rat/Norway rat). In the USA, the seroprevalences of anti-HEV antibodies were 76.9% (83/108 samples tested positive) between 1986 and 1997, 68.5% (135/197) between 1994 and 1998, and 73.5% (144/196) between 2005 and 2006, considerably higher than in other countries [17,18,21]. In Japan, the rates were 31.5% (114/362) from 2000 through 2002, while another study with unreported sample collection dates resulted in 28.6% seroprevalence (16/56) [22,23]; in Germany, the seroprevalence was 24.5% (36/147) between 2007 and 2010 [24], comparable to China with 27.8% (64/230) between 2011 and 2012 [25]. In Vietnam, two separate studies revealed similar seroprevalences of 20.3% (25/123) and 22.3% (21/94) in 2011 and an additional study between 2012 and 2013 demonstrated a lower seroprevalence of 12.3% (48/389) [26,27,28]. In a recent study in Lithuania, the seroprevalence was 31.2% (34/109) between 2014 and 2017; however, data were derived from both Norway rats (27) and Black rats (82) and individual species rates were not indicated [29].

Six studies have been carried out in *Rattus rattus* (Black rat) with results varying considerably. In the USA, one study reported a seroprevalence of 90.2% (102/113) between 1986 and 1997, yet only 38.3% (31/81) between 1994 and 1998 in another [17,18]. In Indonesia, three studies have been conducted with positive rates of 18.1% (21/116) between 2011 and 2012, 37.1% (137/369) in 2012, and 4.1% (10/242) between 2014 and 2016 [30,31,32]. In Japan, seroprevalence was 13.3% (12/90) from 2000 through 2002 [22].

Three studies in Vietnam found anti-HEV seroprevalences in *Rattus tanezumi* (Oriental house rat) of 25.0% (4/16) and 33.3% (2/6) in 2011 and 19.6% (9/46) between 2012 and 2013 [26,27,28]. In a large-scale investigation of sera of wild rats in China between 2011 and 2012, anti-HEV IgG rates in the species *Rattus rattoides losea* (Losea rat), *Rattus flavipectus* (Yellow-breasted rat), *Rattus rattus hainanus*, and *Bandicota indica* (Greater bandicoot rat), were 21.5% (26/121), 19.9% (34/171), 11.8% (2/17), and 23.0% (40/174), respectively [25]. In India, prevalence of anti-HEV Immunoglobulin G (IgG) was 15.8% (9/57) in 1985 in *Rattus rattus rufescens* (Indian black rat), 3.6% (2/55) in 1990 in *Rattus rattus andamanesis* (Sikkim rat), and 54.5% (12/22) in 1985 in *Bandicota bengalensis* (Lesser bandicoot rat) [33]. In the USA, two species within the family *Muridae*, *Rattus exulans* (Polynesian rat) and *Mus musculus* (House rat), had seroprevalences of 83.3% (15/18) between 1986 and 1997 and 14.3% (2/14) between 1994 and 1998, respectively [17,18]. Lastly, a study conducted in Brazil in 2005 found two of four wild rats (species unknown) were positive for anti-HEV IgG [34].

Antibodies against HEV were also found in multiple rodent species within the family *Cricetidae*. Seroprevalence rates in *Clethrionomys gapperi* (Southern red-backed vole), *Neotoma albigula* (White-throated woodrat), *Neotoma mexicana* (Mexican woodrat), *Neotoma micropus* (Southern plains woodrat), *Oryzomys palustris* (Marsh rice rat), *Peromyscus boylei* (Brush mice), *Peromyscus eremicus* (Cactus mouse), *Peromyscus leucopus* (White-footed mouse), *Peromyscus maniculatus* (North American deermouse), and *Sigmodon hispidus* (Hispid cotton rat) were 66.7 (4/6), 59.1 (13/22), 57.1 (48/84), 12.5 (1/8), 24.4 (10/41), 8.3 (2/24), 42.9 (3/7), 10.0 (5/50), 11.0 (11/91), and 32.7 (37/113), respectively. Rodent sera were collected during 1994–1998 throughout the USA and assessed by two in-house established serological tests including antigens based on a mosaic protein composed of recombinant HEV ORF2 and ORF3 and a 55-kDa HEV ORF2 protein [18].

Additionally, seroprevalence in *Suncus murinus* (House shrew) within the family *Soricidae* of the order Soricomorpha was 10.4% (27/260) for anti-HEV IgG and 4.6% (12/260) for Immunoglobulin M (IgM), respectively, with samples collected between 2011 and 2012 in China [35]. The studies in terms of seroprevalence of anti-HEV antibodies are summarized in Table 1.

Differing seroprevalence of anti-HEV antibodies may be due to different serological tests, which have been carried out using commercial anti-HEV enzyme-linked immunosorbent assay (ELISA) kits or conducted with in-house established assays based on diverse antigens (truncated or complete human and rat HEV ORF2 protein) in numerous labs independently. Furthermore, although there is only a single serotype of HEV, the cross-reaction of other known or unknown viral pathogens cannot be excluded and may impact the results. Finally, no HEV seroprevalence studies have been performed on species of the orders Carnivora and Falconiformes.

### 2.2. Detection of Orthohepevirus C Genomes

Molecular biology methods, including RT-PCR amplification, Sanger sequencing and metagenomic analysis with next-generation sequencing, have facilitated the detection of *Orthohepevirus* C (previously designated rat HEV) RNA from liver, feces, and blood specimens of various animal species. Furthermore, comprehensive sequence and phylogenetic analyses enable genotyping of viral strains. Consequently, an increasing number of novel *Orthohepevirus* C variants have been discovered around the world whose assignment remain to be determined.

*Orthohepevirus* C sequences were initially detected in Germany in the widespread rat species *Rattus norvegicus* (Brown rat/Norway rat) [19,20]. To date, the vast majority of *Orthohepevirus* C genomes have been identified in Norway rats, from countries including the USA, China, Germany, France, Denmark, Lithuania, England, Hungary, Austria, Switzerland, Italy, Spain, Greece, Belgium, the Czech Republic, and Vietnam, with all viral strains belonging to HEV-C1 [19,20,24,25,29,36,37,38,39,40,41,42,43,44,45,46]. *Orthohepevirus* C genomes have been detected in numerous other members of the family *Muridae*: HEV-C1 from *Rattus rattus* (Black Rat) has been detected in Indonesia, China, Kenya, and 12 other European countries comprising Lithuania, Germany, Hungary, Denmark, Austria, Switzerland, France, Italy, Spain, Greece, Belgium, and the Czech Republic [29,30,32,41,47,48]; *Rattus tanezumi* (Oriental house rat) in Vietnam and China have also been reported to harbor the virus [26,36,44,49]; in China, HEV-C1 RNA has been detected in *Rattus losea* (Losea rat), *Rattus flavipectus* (Yellow-breasted rat), and *Bandicota indica* (Greater bandicoot rat) [25,43,44]; *Apodemus chevrieri* (Chevrier’s field mouse) and *Apodemus agrarius* (Striped field mouse) were positive for *Orthohepevirus* C detection; however, these variants are phylogenetically divergent from known HEV-C1 or HEV-C2, and thus, cannot yet be classified [36,50].

Consistent with serological studies, *Orthohepevirus* C RNA was found in several species within other rodent families, including *Cricetidae*, containing *Eothenomys melanogaster* (Père David’s vole), *Eothenomys Inez* (Inez’s red-backed vole), *Myodes rufocanus* (Grey red-backed vole), *Microtus gregalis* (Narrow-headed vole), *Cricetulus migratorius* (Gray dwarf hamster), and *Cricetulus barabensis* (Striped dwarf hamster) from China, *Microtus arvalis* (Common vole) from Hungary and Germany, *Myodes glareolus* (Bank vole) from Germany, and *Necromys Lasiurus* (Hairy-tailed bolo mouse) and *Calomys tener* (Delicate vesper) from Brazil [36,50,51,52,53]. The assignment of these newly identified HEV variants remains unclear.

Additionally, *Orthohepevirus* C sequences have been detected in other animal species of mammals. Within the family *Soricidae* of the order of Soricomorpha, the species *Suncus murinus* (House shrew/Asian musk shrew) harbor HEV-C1 in China and Asian musk shrew have been implied as a reservoir for HEV-C1 from wild rats, while the virus in the species *Crocidura olivieri* (Olivier’s shrew) from Kenya cannot be assigned due to increased genetic divergence [35,44,48,50].

In the order Carnivora, HEV-C1 has been identified in a Syrian brown bear (species *Ursus arctos syriacus*, family *Ursidae*) in a German zoo, most likely the result of a spillover infection from free-roaming Norway rats [42]. In 2012, HEV-C2 was firstly reported in Western polecats (species *Mustela putorius*) from the Netherlands [54]. Soon afterwards, HEV-C2 was detected in ferrets from the USA, Japan, and China and in minks from Denmark and China [54,55,56,57]. Both species *Mustela putorius* and *Neovison vison* belong to the carnivore family *Mustelidae*. Even though a short (362 nucleotide) partial ORF1 sequence was identified in a red fox (species *Vulpes vulpes,* family *Canidae*), classification into *Orthohepevirus* C requires comparisons based upon complete genome sequences, as recommended by the International Committee on the Taxonomy of Viruses (ICTV) *Hepeviridae* Study group [58].

Recently, novel HEV variants have been reported from common kestrel (species *Falco tinnunculus*) and red-footed falcon (species *Falco vespertinus*) within the family *Falconidae* of the order Falconiformes in Hungary, notably the viral strain from kestrel showed higher sequence similarity to members of *Orthohepevirus* C than to *Orthohepevirus* B and clustered together with aforementioned rodent HEV variants from China and Brazil; therefore, the kestrel-derived HEV strain is included in this review [59].

Finally, nine HEV-C1 strains have been reported in humans, eight being typical rat HEV sequences from Hong Kong and the other divergent rat HEV sequence derived possibly from Uganda [14,15,16]. However, the precise source and transmission pattern of these nine HEV-C1 strains remain unclear. The studies with regards to the detection of *Orthohepevirus* C genomes are listed in Table 2.

## 3. Genomic Characterization of *Orthohepevirus* C

**Figure 2:**
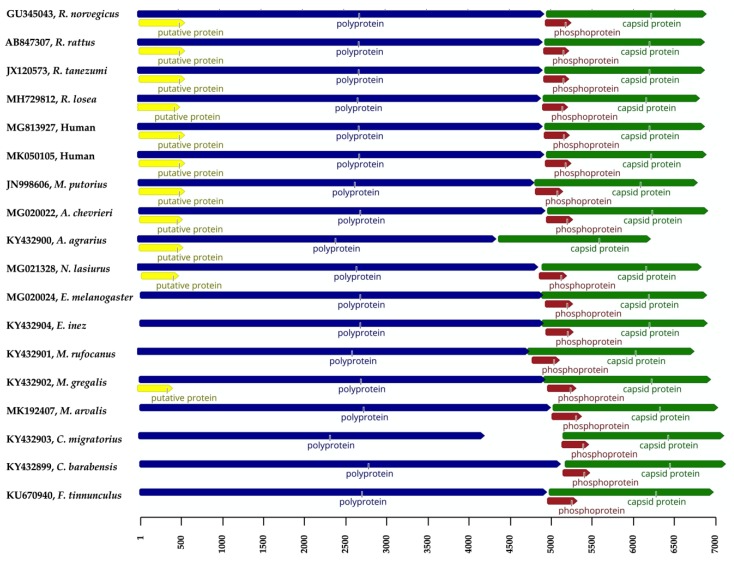
Schematic description of genomic organization of *Orthohepevirus* C variants. Virus designations including GenBank accession numbers and host information are indicated on the left. HEV open reading frames (ORFs) 1, 2, 3, and the putative ORF4 are represented in blue, green, brown, and yellow, respectively. The scale is in bases.

Despite the detection of *Orthohepevirus* C RNA in numerous species, the vast majority of deposited sequences consist of partial HEV ORF1 or ORF2 genes. Complete genomic sequences of *Orthohepevirus* C have been obtained from various mammal species, including *Rattus norvegicus*, *Rattus rattus*, *Rattus tanezumi*, *Rattus losea*, *Apodemus chevrieri*, *Apodemus agrarius*, *Eothenomys melanogaster*, *Eothenomys inez*, *Myodes rufocanus*, *Microtus gregalis*, *Microtus arvalis*, *Necromys lasiurus*, *Cricetulus migratorius*, *Cricetulus barabensis*, *Mustela putorius*, *Falco tinnunculus*, and human (Table 1). For a better comparative analysis and genomic characterization of *Orthohepevirus* C, the schematic description of genomic organization of representative *Orthohepevirus* C variants from individual animal species is depicted in Figure 2.

Overall, the genomic length of *Orthohepevirus* C sequences ranges from 6.8 to 7.2 kb, excluding those without sequenced 5′ or 3′ ends. However, the distinct strain RtAa-HEV/JL2014 (GenBank accession no. KY432900) from *Apodemus agrarius*, which consists of only 6286 nucleotides and has a significant shorter ORF1 with 4334 nucleotides; moreover, RtAa-HEV/JL2014 lacks ORF3. Since it has been recently reported that the ORF3 is essential for the release of membrane-associated rat HEV particles, the absence of ORF3 in *Apodemus agrarius* needs further verification [60]. In addition to RtAa-HEV/JL2014, the ORF1 of the strain RtCm-HEV/XJ2016 (GenBank accession no. KY432903) from *Cricetulus migratorius* is only 4182 nucleotides in length. However, the short viral genome and ORF1, as well as the absence of ORF3 may be associated with errors in assembling reads to contigs and mapping assembled contigs to references in the metagenomic study [36].

A unique ORF (tentatively named ORF4), located within the 5′ region of ORF1 has been proposed in certain rat and ferret HEV strains [32,55]. As shown in Figure 2, all the HEV-C1 strains from the species including *Rattus norvegicus*, *Rattus rattus*, *Rattus tanezumi*, and *Rattus losea* within the family *Muridae*, two HEV-C1 analogues identified from humans, HEV-C2 strains from the family *Mustelidae*, and the HEV variants from the species *Necromys lasiurus* and *Microtus gregalis* within the family *Cricetidae* harbor ORF4. In contrast, this putative ORF is not found in other species. A recent study has shown that ORF4 is not necessary for the active replication of rat HEV [60]. Therefore, the functional role of the putative ORF4 remains poorly understood. Furthermore, due to the significant genetic variability within *Orthohepevirus* C, the molecular evolution of ORF4 is as of yet unclear.

Typically, HEV ORF2 overlaps partially with ORF3 but not ORF1. However, three HEV variants in the species *Eothenomys melanogaster*, identified from two separate studies, possess ORF2 genes overlapping with ORF1. This is also the case for HEV strains in the species *Eothenomys inez*, *Myodes rufocanus*, and *Microtus gregalis* within the family *Cricetidae* [8,36,50]. Additionally, the unique motif at the 5‘-untranslated regions containing the 10 nucleotides GCAACCCCG is exclusively present in HEV variants of the family *Muridae* [31]. Finally, *Orthohepevirus* C variants utilize distinct translational frames, as described in our recent study [43].

Although many novel HEV variants within the *Orthohepevirus* C species have been discovered in diverse wild rodents in China in a study based on metagenomic analysis, each species has only a single viral complete genomic sequence [36]. The genomic features and genetic heterogeneity of these HEV variants in individual animal species awaits the availability of more complete genome sequences.

## 4. Genetic Variability of *Orthohepevirus* C

As mentioned, the majority of described *Orthohepevirus* C sequences are partial genomic fragments (<500 bp). In order to determine the genetic variability of HEV variants within the species *Orthohepevirus* C, we have included 48 currently available complete or nearly complete genomic sequences of *Orthohepevirus* C from GenBank. The pairwise comparisons of complete genome and deduced ORFs of *Orthohepevirus* C is illustrated in Table 3.

Unexpectedly, the genomic comparisons between HEV-C1 and other newly identified HEV strains derived from the rodent species *Apodemus chevrieri*, *Apodemus agrarius*, *Eothenomys melanogaster*, *Eothenomys inez*, *Myodes rufocanus*, *Microtus gregalis*, *Microtus arvalis*, *Necromys lasiurus*, *Cricetulus migratorius*, and *Cricetulus barabensis* exhibit comparable or even higher divergence than is present between HEV-C1 and HEV-C2, even though HEV-C2 strains have only been found in the carnivore species *Mustela putorius*. Nucleotide identities in *Orthohepevirus* C variants from individual rodent species vary between 48.2–67.3%. Higher identities are observed between *Eothenomys melanogaster* and *Eothenomys Inez,* ranging from 72.2 to 72.6%, and from 76.0 to 77.3% between *Microtus gregalis* and *Microtus arvalis*. Similar results have been observed for the nucleotides and amino acids of the 3 coding regions. Notably, for the comparison of ORF3, most of the HEV variants in their respective group are highly divergent and exhibit low identities, especially at amino acid level (<20%). These data highlight the fact that substantial genetic variability of HEV is present within the order Rodentia, raising a need for increased efforts to characterize the variants circulating in the respective habitats. Only through this will we be able to better assess the risk potential of these viruses to animal and human health.

Due to the high identity (> 75%) to HEV-C1 strains, two HEV variants detected in humans in Hong Kong and Uganda can be considered as HEV-C1 [14,15]. Furthermore, the kestrel HEV strain identified in Hungary shares relatively high similarity (> 83%) to the HEV strains from the species *Microtus arvalis* from Hungary, Germany, and the Czech Republic, which is comparable to sequence identities within common vole variants (between 81.5% and 91.9%). Therefore, it is assumed that the detection of HEV variants in birds of prey (Common kestrel and Red-footed falcon) may be linked to the consumption of wild rodents, in particular common voles [52,53,59].

## 5. Molecular Evolution of *Orthohepevirus* C

**Figure 3:**
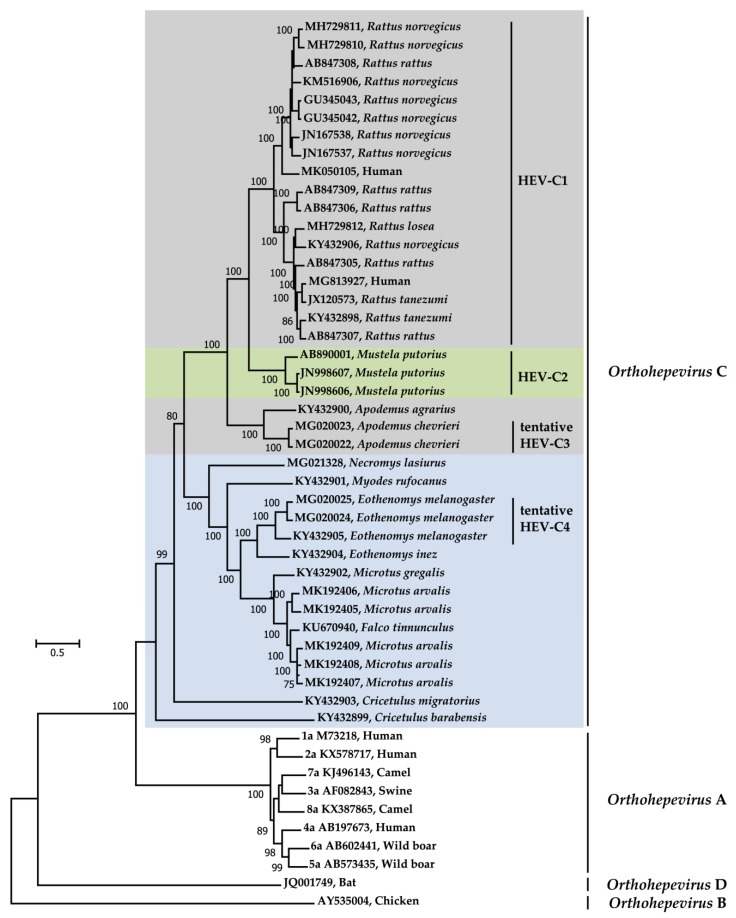
Maximum-likelihood phylogeny of *Orthohepevirus* C variants within the genus *Orthohepevirus*. Representatives of *Orthohepevirus* A, B, and D variants are reference strains according to the International Committee on Taxonomy of Viruses (ICTV) consensus proposals [11]. Virus designations include GenBank accession numbers and host information. Species and genotypes are indicated on the right. Viral strains from rodent families *Muridae* and *Cricetidae* and carnivore family *Mustelidae* are highlighted with grey, blue, and green background, respectively. Complete genome sequences were aligned using the MAFFT algorithm. Phylogenetic reconstructions were performed using MEGA version 7 with 1000 bootstrap replicates. General Time Reversible (GTR) + Gamma Distributed (G) + Invariable Sites (I) nucleotide substitution model was selected based on Find Best-Fit Substitution Model (ML) in MEGA version 7. Bootstrap values (>75%) are indicated at specific nodes. Scale bars indicate the number of nucleotide substitutions per site.

In order to investigate the dynamics of the molecular evolution of *Orthohepevirus* C, phylogenetic analysis was conducted using representative complete genomic sequences within the genus *Orthohepevirus* of the *Hepeviridae* family (Figure 3). The discrimination of genotypes within the species *Orthohepevirus* A and C is in accordance with consensus proposals for the classification from ICTV *Hepeviridae* study group [11]. Recently, we have reported novel HEV variants in Chevrier’s field mouse (species *Apodemus chevrieri*, family *Muridae*) and Père David’s vole (species *Eothenomys melanogaster*, family *Cricetidae*) in China and two viral genomes of each animal species were amplified and characterized. Due to the significant divergence from HEV-C1 and HEV-C2, we have proposed these variants be classified as putative HEV-C3 and putative HEV-C4, which has been included and discussed in several recent research articles [13,50,53]. Afterwards, highly diverse HEV variants have been discovered in other wild rodent species around the world [36,51,53]. Collectively, phylogenetic tree shows that *Orthohepevirus* C variants form a separate branch within the genus *Orthohepevirus.* Predictably, HEV variants detected in the rodent genus *Rattus* and the carnivore species *Mustela putorius* cluster into two independent clades within the species *Orthohepevirus* C, which have already been defined as HEV-C1 and HEV-C2, respectively. Even though newly identified HEV variants are phylogenetically separate from HEV-C1 and HEV-C2, HEV variants from the rodent genus *Apodemus* (including species *Apodemus agrarius* and *Apodemus chevrieri*), *Eothenomys* (including species *Eothenomys melanogaster* and *Eothenomys Inez*), and *Microtus* (including the species *Microtus gregalis* and *Microtus gregalis*) clustered into three distinct clusters, which are associated with rodent taxonomy and host specificity. Taken together, it is hypothesized that co-evolutionary model, and not the host-switch pattern, might be applied regarding the species *Orthohepevirus* C [10]. However, the HEV variants derived from the rodent family *Cricetidae*, particularly RtCm-HEV/XJ2016 (GenBank accession no. KY432903) from the species *Cricetulus migratorius*) and RtCb-HEV/HeB2014 (GenBank accession no. KY432899) from *Cricetulus barabensis*, demonstrate a high degree of divergence within the *Orthohepevirus* C clade. With no doubt that the evolutionary history of *Orthohepevirus* C will be better elucidated when diversified HEV strains are discovered in insectivores and carnivores in the future.

The two HEV variants derived from a Chinese and Canadian patient, respectively, cluster within the HEV-C1 branch, providing further evidence of a potential zoonotic origin [14,15]. Concordant with pairwise comparisons of complete viral genomes and coding regions, the HEV strain (GenBank accession no. KU670940) identified in a common kestrel (*Falco tinnunculus*) from Hungary clusters together with the five reported HEV variants from European common voles (*Microtus arvalis*). Whether *Orthohepevirus* C variants from common voles indeed can infect common kestrel remains to be determined [52,53,59].

Thus far, all HEV variants detected in rodents have been isolated from the families *Muridae* and *Cricetidae*. Considering that the order Rodentia comprises 33 families and 2277 species (>40% of all mammal species), therefore forming the largest mammalian order, we believe that these HEV variants are only the tip of the iceberg. Future studies will undoubtably reveal further novel variants within *Orthohepevirus* C with new *Orthohepevirus* C genotypes being assigned in the coming years. Although it is evident from our analyses that HEV variants within each genotype of *Orthohepevirus* C (including HEV-C1, HEV-C2 and other unassigned groups) are highly divergent from one another, subtype differentiation has not yet been proposed by ICTV *Hepeviridae* study group [11,31,53].

It is a well-known fact that evolution of HEV within the species *Orthohepevirus* A undergoes different patterns between different genotypes (HEV-1 to HEV-8), which is associated with at least two distinct epidemiological profiles of the species [61]. Increasingly genetically diversified viruses within *Orthohepevirus* C have been identified; to what extent of viral evolutionary discrepancy of the species is largely unclear.

## 6. Cross-Species Transmission of *Orthohepevirus* C

Human hepatitis E cases in industrialized countries are mainly caused by zoonotic transmission of HEV-3 and HEV-4 within the species *Orthohepevirus* A; domestic swine, wild boar, and sika deer have been confirmed to be the reservoirs. Typically, HEV zoonotic transmission occurs via three routes. Firstly, population groups coming into direct contact with infected animals, such as veterinarians and workers on pig farms or slaughterhouses, who were proven to have significantly higher anti-HEV IgG seroprevalence rates than the general population and other veterinarians in some European countries; secondly, foodborne transmission via consumption of undercooked infectious meat products (mostly pork), such as sausages and liver, which is common in European countries; and thirdly, environmental contamination by animal feces, since the use of pig manure in agriculture may lead to contamination of water sources and agricultural products [3]. Furthermore, consumption of food products (milk and meat) from camels and subsequent infection with a camelid HEV-7 strain was implicated in a case of chronic hepatitis E after transplantation in a patient from the United Arab Emirates [62]. Using reverse genetic systems, the potential of zoonotic infection of HEV-7 as well as HEV-5 from wild boar have been confirmed by experimentally infecting primates in Japan [63,64]. Recently, one study has demonstrated that HEV-8 from Bactrian camels in multiple regions in China was able to cause chronic hepatitis in cynomolgus macaques (human surrogate), indicating a high risk of zoonosis [65]. Lastly, rabbit HEV-3ra might also pose a potential threat to humans, based on described cross-species infection of pigs and cynomolgus macaques [66,67].

Rodents are extraordinarily abundant and diversified in multiple continental habitats and live in close proximity to humans, domestic animals, and wild animals. This interface has resulted in the rodent origin of a plethora of zoonotic viruses, such as Lassa virus, Lymphocytic choriomeningitis virus, Sin Nombre virus, Hantaan virus, and Seoul virus, all of which have caused severe diseases in humans in the past decades [68]. Increasing amounts of evidence suggest that rodents may also play a role in the spread and epidemiology of HEV. Numerous studies from different countries have reported that rodent sera tested positive for anti-HEV antibodies were able to cross-react with human-derived HEV antigens [17,18,21,22,23]. Initially, a 1996 study from Thailand reported experimental infection of three Wistar laboratory rats with a human pathogenic HEV strain derived from patient feces acquired during a hepatitis E outbreak in Nepal in 1992 [69]. Later, a Chinese study demonstrated the successfully infection of Balb/c nude mice with swine HEV and active infection of Sprague-Dawley after intrahepatic inoculation with infectious HEV-4 RNA [70]. A German study reported detectable HEV RNA and anti-HEV antibodies in Wistar rats inoculated with a wild boar-derived HEV-3 strain [71]. In addition, multiple rodent cell lines have been successfully infected with HEV-3 [72]. Finally, zoonotic HEV-3 was reported in wild rats in the USA, England, and Japan, and a rabbit HEV-3ra sequence was detected in a Norway rat from Belgium. However, viral RNA was mostly detected in intestines and feces and seldom in the liver in these cases [23,41,46]. In contrast, the results from a series of studies demonstrated that rats are susceptible to rat HEV but not HEV-1, HEV-2, or HEV-4 [37,73]. Therefore, the question whether rodents are truly natural reservoirs of human HEV or act as only intermediate hosts is not yet conclusively answered.

Although rodents have been confirmed to be the primary natural reservoirs of HEV variants within the species *Orthohepevirus* C, the potential risk of these viruses concerning cross-species infection and zoonotic transmission to humans remains a controversial topic [13]. In the past, *Orthohepevirus* C variants were understudied and neglected due to their genetic divergence (identities <52%) to the principle human pathogenic HEV strains (HEV-1 to HEV-4). It has been reported that pigs or non-human primates (rhesus monkeys) inoculated with rat HEV showed no evidence of infection, indicating that it is not a source of human infection [37,66]. In contrast, after screening with ELISAs based on the capsid protein of Norway rat HEV, several sera from forestry works of eastern Germany were shown to have strong reactivity [74]. In a recent study from Vietnam, IgG antibody titers against HEV-C1 antigen in three of the 99 sera of hospitalized febrile patients were more than eight-fold higher than those against HEV-1 antigen, while IgM antibodies against HEV-C1 antigen were detected in acute sera from two of the three patients using both ELISA and Western blotting and one patient developed illness with mild liver dysfunction [75]. Furthermore, in Japan, a rat HEV reverse genetics system was demonstrated to be replication competent in a human cell line, while another study reported that rat HEV from *Rattus rattus* could propagate efficiently in human hepatoma cell lines including PLC/PRF/5, HuH-7, and HepG2 [76]. Intriguingly, it was reported that a typical rat HEV caused persistent hepatitis in a liver transplant patient in Hong Kong and that the virus was cleared after ribavirin treatment. Additionally, a divergent rat HEV from Uganda induced severe acute hepatitis in an immunocompetent patient from Canada. These two case reports provide first evidence of a possible zoonotic potential of rat HEV [14,15]. Furthermore, based on comprehensive clinical and epidemiological analyses, seven rat HEV infections have been further identified in Hong Kong, and these strains are extremely close to an isolate from a rat captured near the residences of patients. Remarkably similar to *Orthohepevirus* A, rat HEV have caused chronic viral infection in immunosuppressed individuals as well as extrahepatic manifestations [16]. Jointly, it is highly presumable that rat HEV may be a cause of human hepatitis. Nevertheless, there are still many open questions regarding zoonotic transmission of *Orthohepevirus* C. What is the viral determinant for rat HEV cross-species infection? What is the effectivity of antiviral therapy, especially regarding ribavirin, against rat HEV infection? Will other rat HEV analogues (e.g., newly identified HEV strains from wild rodents, insectivores, and carnivores) be capable of infecting humans?

Interspecies transmission of *Orthohepevirus* C variants has been characterized in several studies as well. House shrews (*Suncus murinus*) have been reported as a reservoir of rat HEV worldwide; however, a novel divergent HEV strain has been detected in a Olivier’s shrew (*Crocidura olivieri*) from Kenya and formed a separate monophyletic branch from HEV-C1, indicating the relationship between the viral species *Orthohepevirus* C and the animal order Soricomorpha should be more complex than previously considered [35,43,44,48]. Although ferret HEV (HEV-C2) can cause both acute and persistent infection in ferrets, researchers failed to infect monkeys and rats with ferret HEV [77].

## 7. Conclusions and Outlook

An increasing number of HEV variants are being detected in diverse animal species, including mammals, avian, and fish, with some of these species having been confirmed as natural reservoirs for HEV and sources of zoonotic infection. With the advancement of techniques such as high-throughput sequencing in metagenomics studies, no doubt more novel HEV variants will be discovered in the near future. The detection of numerous emerging HEV variants within the species *Orthohepevirus* C has expanded the known host range of HEV dramatically. This raises critical concerns about the potential risk of cross-species infection and zoonotic transmission to humans, as the biology, ecology, natural history, and pathogenesis of these viruses are largely unknown. After three recent reports identified totally nine human hepatitis E cases linked to rat HEV, the necessity to broaden our knowledge concerning the molecular epidemiology of circulating *Orthohepevirus* C variants is clear, and the potential risks of these viruses to public health should be reassessed. To elucidate the zoonotic potential of *Orthohepevirus* C variants, the patterns of transmission, mechanisms of replication, and functional roles of proteins merit further investigation.

## Figures and Tables

**Table 1:** Seroprevalence of anti-HEV antibodies.

Order	Family	Species (Scientific Name)	Common Name ^1^	No. Positive/No. Tested (%) ^3^	Sampling Site (s) (Year)	Assay	Reference
Rodentia	*Muridae*	*Rattus norvegicus*	Brown rat/Norway rat	83/108 (76.9)	The USA (1986–1997)	In-house anti-HEV ELISA	[17]
				135/197 (68.5)	The USA (1994–1998)	In-house anti-HEV ELISAs	[18]
				144/196 (73.5)	The USA (2005–2006)	In-house anti-HEV ELISA	[21]
				114/362 (31.5)	Japan (2000–2002)	In-house anti-HEV ELISA	[22]
				16/56 (28.6)	Japan (N.A.)	Viragent HEV-Ab kit	[23]
				36/147 (24.5)	Germany (2007–2010)	In-house anti-rat HEV ELISA	[24]
				64/230 (27.8)	China (2011–2012)	In-house anti-rat HEV ELISA	[25]
				25/123 (20.3)	Vietnam (2011)	In-house anti-rat HEV ELISA	[26]
				21/94 (22.3)	Vietnam (2011)	In-house anti-rat HEV ELISA	[27]
				48/389 (12.3)	Vietnam (2012–2013)	In-house anti-rat HEV ELISA	[28]
				34/109 (31.2) ^4^	Lithuania (2014–2017)	In-house anti-rat HEV ELISA	[29]
		*Rattus rattus*	Black rat	102/113 (90.2)	The USA (1986–1997)	In-house anti-HEV ELISA	[17]
				31/81 (38.3)	The USA (1994–1998)	In-house anti-HEV ELISAs	[18]
				21/116 (18.1)	Indonesia (2011–2012)	In-house anti-HEV ELISA	[30]
				137/369 (37.1)	Indonesia (2012)	In-house anti-rat HEV ELISA	[31]
				10/242 (4.1)	Indonesia (2014–2016)	In-house anti-rat HEV ELISA	[32]
				12/90 (13.3)	Japan (2000–2002)	In-house anti-HEV ELISA	[22]
		*Rattus tanezumi*	Oriental house rat	4/16 (25.0)	Vietnam (2011)	In-house anti-rat HEV ELISA	[26]
				2/6 (33.3)	Vietnam (2011)	In-house anti-rat HEV ELISA	[27]
				9/46 (19.6)	Vietnam (2012–2013)	In-house anti-rat HEV ELISA	[28]
		*Rattus rattoides losea*	Losea rat	26/121 (21.5)	China (2011–2012)	In-house anti-rat HEV ELISA	[25]
		*Rattus flavipectus*	Yellow-breasted rat	34/171 (19.9)	China (2011–2012)	In-house anti-rat HEV ELISA	[25]
		*Rattus rattus hainanus*	N.A. ^2^	2/17 (11.8)	China (2011–2012)	In-house anti-rat HEV ELISA	[25]
		*Bandicota indica*	Greater bandicoot rat	40/174 (23.0)	China (2011–2012)	In-house anti-rat HEV ELISA	[25]
		*Rattus rattus rufescens*	Indian black rat	9/57 (15.8)	India (1985)	In-house anti-HEV ELISA	[33]
		*Rattus rattus andamanesis*	Sikkim rat	2/55 (3.6)	India (1990)	In-house anti-HEV ELISA	[33]
		*Bandicota bengalensis*	Lesser bandicoot rat	12/22 (54.5)	India (1985)	In-house anti-HEV ELISA	[33]
		*Rattus exulans*	Polynesian rat	15/18 (83.3)	The USA (1986–1997)	In-house anti-HEV ELISA	[17]
		*Mus musculus*	House rat	2/14 (14.3)	The USA (1994–1998)	In-house anti-HEV ELISAs	[18]
	*Cricetidae*	*Clethrionomys gapperi*	Southern red-backed vole	4/6 (66.7)	The USA (1994–1998)	In-house anti-HEV ELISAs	[18]
		*Neotoma albigula*	White-throated woodrat	13/22 (59.1)	The USA (1994–1998)	In-house anti-HEV ELISAs	[18]
		*Neotoma mexicana*	Mexican woodrat	48/84 (57.1)	The USA (1994–1998)	In-house anti-HEV ELISAs	[18]
		*Neotoma micropus*	Southern plains woodrat	1/8 (12.5)	The USA (1994–1998)	In-house anti-HEV ELISAs	[18]
		*Oryzomys palustris*	Marsh rice rat	10/41 (24.4)	The USA (1994–1998)	In-house anti-HEV ELISAs	[18]
		*Peromyscus boylei*	Brush mice	2/24 (8.3)	The USA (1994–1998)	In-house anti-HEV ELISAs	[18]
		*Peromyscus eremicus*	Cactus mouse	3/7 (42.9)	The USA (1994–1998)	In-house anti-HEV ELISAs	[18]
		*Peromyscus leucopus*	White-footed mouse	5/50 (10.0)	The USA (1994–1998)	In-house anti-HEV ELISAs	[18]
		*Peromyscus maniculatus*	North American deermouse	11/91 (11.0)	The USA (1994–1998)	In-house anti-HEV ELISAs	[18]
		*Sigmodon hispidus*	Hispid cotton rat	37/113 (32.7)	The USA (1994–1998)	In-house anti-HEV ELISAs	[18]
Soricomorpha	*Soricidae*	*Suncus murinus*	House shrew	27/260 (10.4)	China (2011–2012)	In-house anti-rat HEV ELISA	[35]

^1^ Taxonomy and common names refer to https://www.iucnredlist.org/. ^2^ N.A. stands for not available. ^3^ Seroprevalence indicates presence of anti-HEV IgG. ^4^ Positive rate derived from both *Rattus norvegicus* and *Rattus rattus*, no individual species rates reported. HEV, hepatitis E virus; ELISA, enzyme-linked immunosorbent assay.

**Table 2:** Detection of *Orthohepevirus* C genomes.

Order	Family	Species (Scientific Name)	Common Name ^1^	Sampling Site (s) (Year)	Virus Genotype	Genomic Sequence	Reference
Rodentia	*Muridae*	*Rattus norvegicus*	Brown rat/Norway rat	The USA (2003), Germany (2007–2010), Germany (2009–2016), France (2011–2012), Denmark (2012), China (2011–2012; 2013–2016; 2014–2017), Lithuania (2014–2017), 11 European countries (2005–2016) ^2^, England (2014–2016), Vietnam (2011; 2012–2013), Museum collections ^3^	HEV-C1	Partial, Nearly complete ^5^, Complete	[19,20,24,25,28,29,36,37,38,39,40,41,42,43,44,45,46]
		*Rattus rattus*	Black rat	Indonesia (2011–2012; 2014–2016), Lithuania (2014–2017), Italy (2015), 11 European countries (2005–2016), Kenya (2016); Museum collections	HEV-C1	Partial, Complete	[29,30,32,41,47,48]
		*Rattus tanezumi*	Oriental house rat	Vietnam (2011; 2012–2013), China (2013–2016; 2014–2017)	HEV-C1	Partial, Complete	[26,28,36,44,49]
		*Rattus losea*	Losea rat	China (2011–2012; 2014–2017)	HEV-C1	Partial, Nearly complete	[25,44]
		*Rattus flavipectus*	Yellow-breasted rat	China (2011–2012)	HEV-C1	Partial	[25,43]
		*Bandicota indica*	Greater bandicoot rat	China (2011–2012)	HEV-C1	Partial	[25]
		*Apodemus chevrieri*	Chevrier’s field mouse	China (2013–2015)	N.A. ^4^	Partial, Complete	[50]
		*Apodemus agrarius*	Striped field mouse	China (2013–2016)	N.A.	Complete	[36]
	*Cricetidae*	*Eothenomys melanogaster*	Père David’s vole	China (2013–2015)	N.A.	Partial, Complete	[36,50]
		*Eothenomys inez*	Inez’s red-backed vole	China (2013–2016)	N.A.	Complete	[36]
		*Myodes rufocanus*	Grey red-backed vole	China (2013–2016)	N.A.	Nearly complete	[36]
		*Microtus gregalis*	Narrow-headed vole	China (2013–2016)	N.A.	Complete	[36]
		*Cricetulus migratorius*	Gray dwarf Hamster	China (2013–2016)	N.A.	Complete	[36]
		*Cricetulus barabensis*	Striped dwarf hamster	China (2013–2016)	N.A.	Complete	[36]
		*Microtus arvalis*	Common vole	Hungary (2016), Germany and the Czech Republic (2009–2013)	N.A.	Partial, Complete	[52,53]
		*Myodes glareolus*	Bank vole	Germany (2009–2013)	N.A.	Partial	[53]
		*Necromys lasiurus*	Hairy-tailed bolo mouse	Brazil (2008–2013)	N.A.	Nearly complete	[51]
		*Calomys tener*	Delicate vesper mouse	Brazil (2008–2013)	N.A.	Partial	[51]
Soricomorpha	*Soricidae*	*Suncus murinus*	House shrew/Asian musk shrew	China (2011–2012; 2013–2016; 2014–2017)	HEV-C1	Partial	[35,43,44]
		*Crocidura olivieri*	Olivier’s shrew	Kenya (2016)	N.A.	Partial	[48]
Carnivora	*Ursidae*	*Ursus arctos syriacus*	Syrian brown bear	Germany (2011–2016)	HEV-C1	Partial	[42]
	*Mustelidae*	*Mustela putorius*	Western polecat/ferret	The Netherlands (2010), the USA (2013), Japan (2009–2013), China (2016),	HEV-C2	Partial, Complete	[54,55,56]
		*Neovison vison*	American mink	Denmark (2008–2011), China (2016),	HEV-C2	Partial	[56,57]
Falconiformes	*Falconidae*	*Falco tinnunculus*	Common kestrel	Hungary (2014)	N.A.	Partial, Complete	[59]
		*Falco vespertinus*	Red-footed falcon	Hungary (2014)	N.A.	Partial,	[59]
Primates	*Hominidae*	*Homo sapiens*	Human	Hong Kong (2017–2019), Uganda (2017)	HEV-C1	Complete	[14,15,16]

^1^ Taxonomy and common names refer to https://www.iucnredlist.org/. ^2^ Sampling sites include Germany, Hungary, Denmark, Austria, Switzerland, France, Italy, Spain, Greece, Belgium, and the Czech Republic. ^3^ Liver tissue samples from *Rattus norvegicus* and *Rattus rattus* rats from museum collections covering localities primarily in the USA plus additional samples from China, Honduras, Madagascar, Mexico, Nicaragua, Peru, Russia, and Vietnam. ^4^ N.A. stands for not assigned. ^5^ Nearly complete indicates viral genome lacking 5′ end.

**Table 3:** Pairwise comparisons of complete genome and deduced ORFs of *Orthohepevirus* C variants.

*Orthohepevirus* C (No. of Variants) ^1^	% Identity											
	HEV-C1 (20) ^2^	HEV-C2 (10)	RdAcHEV (2)	RdAaHEV (1)	RdEmHEV (3)	RdEiHEV (1)	RdMrHEV (1) ^3^	RdMgHEV (1)	RdMaHEV (6) ^4^	RdNlHEV (1)	RdCmHEV (1)	RdCbHEV (1)
	Complete genome (nt)											
HEV-C1		65.3–67.7	62.6–65.0	56.5–59.1	53.2–55.4	54.3–55.4	53.1–55.2	53.8–55.5	52.9–55.3	54.8–56.0	49.9–51.3	52.6–54.1
HEV-C2	63.5–65.9/**69.5–74.0**		58.5–58.6	62.1–62.8	54.6–55.6	54.7–54.9	53.3–53.7	54.8–55.0	53.1–54.4	54.9–55.1	53.4–53.6	51.1–51.5
RdAcHEV	61.0–63.9/**64.4–67.8**	61.0–61.6/**67.8–68.5**		66.2–66.4	54.3–55.0	54.5–54.7	53.6–53.8	54.5–54.9	53.0–54.1	54.5–54.6	52.7	50.4–51.0
RdAaHEV	54.8–56.5/**57.1–61.1**	56.3–56.6/**61.9–62.1**	63.2–63.5/**72.9–73.6**		51.8–51.9	52.3	51.0	51.6	49.7–50.8	52.0	50.5	48.2
RdEmHEV	51.4–53.4/**49.6–53.5**	53.1–53.7/**52.5–54.3**	51.9–52.9/**51.3–53.2**	49.1–49.5/**47.6–48.6**		72.2–72.6	62.1–63.0	66.9–67.3	65.7–67.2	59.5–59.9	54.5–54.6	52.2–52.5
RdEiHEV	52.7–53.7/**50.0–52.7**	53.1–53.2/**53.3–53.6**	52.6–52.7/**51.8–52.1**	49.6/**48.4**	70.3–70.9/**80.2–82.4**		62.6	67.1	64.9–65.9	60.1	55.0	52.7
RdMrHEV	51.7–53.3/**51.6–54.2**	51.4–52.9/**52.8–53.1**	51.8–52.0/**52.3–52.7**	48.3/**47.9**	60.6–62.0/**67.8–69.1**	61.0/**66.8**		61.4	62.0–62.4	58.3	54.0	52.2
RdMgHEV	52.3–53.8/**50.4–52.9**	53.0–53.1/**52.9–53.8**	52.5–53.0/**51.8–52.1**	49.3/**48.2**	66.2–67.0/**73.0–74.4**	66.4/**73.1**	60.1/**66.0**		76.0–77.3	59.3	54.0	52.8
RdMaHEV	50.9–53.9/**49.6–54.0**	51.1–52.5/**51.7–54.0**	50.8–52.0/**51.2–52.7**	47.4–48.7/**49.4–50.6**	65.1–67.1/**70.9–74.5**	64.0–65.5/**69.9–72.4**	60.5–61.4/**64.6–62.2**	74.0–75.9/**86.6–89.6**		58.9–59.5	53.4–54.2	51.3–52.1
RdNlHEV	53.5–54.9/**53.4–55.8**	54.6/**55.4–55.5**	53.5–53.7/**53.7–53.9**	50.4/**50.5**	59.4–59.8/**60.9–62.3**	59.9/**61.4**	57.5/**61.5**	58.5/**60.9**	57.4–58.7/**59.6–61.0**		53.4	51.4
RdCmHEV	48.0–49.5/**47.5–50.8**	48.1–48.9/**49.7–50.4**	47.7/**49.2–49.4**	44.6/**45.0**	49.3–49.9/**50.1–51.8**	49.6/**50.7**	49.7/**49.9**	49.6/**50.0**	47.6–49.1/**48.4–50.3**	49.6/**50.1**		51.1
RdCbHEV	48.3–49.7/**46.5–48.8**	49.6–49.9/**49.4–50.2**	48.6–49.1/**48.6–48.8**	45.7/**45.2**	50.6–50.8/**48.4–49.6**	49.9/**48.9**	50.5/**48.3**	51.5/**49.1**	49.4–50.5/**46.7–48.0**	49.9/**48.6**	46.1/**45.4**	
	ORF1 (nt/aa)											
	ORF2 (nt/aa)											
HEV-C1		66.5–72.8/**76.4–81.6**	63.2–69.7/**70.7–74.9**	59.6–66.1/**68.2–73.3**	57.0–61.9/**60.7–63.0**	56.8–61.3/**59.7–62.0**	56.5–61.1/**60.2–63.6**	57.2–61.7/**61.2–64.3**	56.6–61.0/**58.7–63.2**	56.6–60.6/**63.0–65.7**	56.6–60.4/**60.1–61.8**	53.1–57.1/**56.4–59.4**
HEV-C2	59.0–63.6/**37.0–45.4**		64.5–65.4/**70.4–71.0**	62.9–63.4/**69.8–70.2**	58.5–60.4/**58.9–59.6**	58.1–58.6/**58.0–59.3**	57.7/**60.9–61.8**	59.6–60.9/**60.6–61.5**	57.7–59.2/**57.7–59.9**	58.9–59.4/**61.3–62.5**	57.9–58.3/**59.3–59.8**	56.7/**56.8–58.1**
RdAcHEV	59.2–62.6/**39.6–50.0**	54.6–56.4/**32.1–35.7**		70.1–70.4/**85.6–85.8**	60.4–61.5/**62.3–62.9**	59.6–60.3/**61.6–61.8**	59.1–59.6/**61.2–61.8**	60.3–60.8/**63.2–63.5**	58.1–60.0/**60.1–61.6**	59.4–59.5/**61.8–62.3**	59.3/**59.2**	55.1–56.3/**57.4–58.0**
RdAaHEV	N.A. ^5^	N.A.	N.A.		58.0–58.4/**60.4–60.7**	58.7/**60.4**	57.4/**59.0**	57.4/**60.2**	55.1–56.4/**57.9–58.7**	57.6/**60.4**	57.1/**57.8**	53.8/**55.6**
RdEmHEV	40.1–44.8/**20.0–27.8**	41.3–46.7/**21.0–26.9**	40.9–44.3/**22.2–28.2**	N.A.		76.5–77.5/**89.0–89.2**	65.4–66.2/**70.6–70.9**	68.9–70.0/**76.3–76.6**	66.6–68.3/**72.1–73.6**	62.6–63.8/**68.4–69.0**	58.0–59.1/**60.3–60.6**	57.3–58.4/**60.2–61.0**
RdEiHEV	42.2–47.5/**20.7–29.3**	40.7–43.6/**20.3–26.3**	42.6–42.9/**23.7–25.4**	N.A.	80.4–81.8/**62.8–67.3**		66.7/**70.9**	69.6/**77.1**	67.8–68.6/**72.7–73.9**	63.9/**69.3**	60.7/**61.4**	59.5/**61.7**
RdMrHEV	37.1–39.4/**19.5–24.6**	37.8–38.1/**19.7–20.5**	35.4–37.1/**22.7**	N.A.	52.0–53.4/**30.8–32.5**	56.2/**39.0**		66.1/**69.7**	65.1–66.2/**67.4–68.6**	63.2/**68.5**	59.0/**60.6**	57.8/**60.9**
RdMgHEV	38.6–41.6/**19.5–24.4**	39.1–41.0/**19.7–22.8**	36.9–38.2/**20.8–21.6**	N.A.	59.4–60.3/**43.7–44.5**	61.1/**44.2**	57.1/**36.3**		78.9–80.2/**90.8–92.1**	64.1/**67.1**	59.9/**62**	57.3/**61.4**
RdMaHEV	36.3–40.2/**10.2–18.9**	36.5–38.9/**10.9–17.8**	37.8–39.5/**10.1–15.5**	N.A.	55.6–59.7/**25.8–31.5**	56.9–61.1/**26.2–31.7**	58.0–61.8/**21.1–26.8**	81.9–82.2/**36.3–41.9**		61.9–62.9/**67.3–67.7**	57.1–58.4/**59.7–60.3**	56.3–57.0/**58.7–59.6**
RdNlHEV	32.9–35.0/**14.9–24.0**	31.7–32.0/**14.4–15.2**	32.9–34.0/**17.2–18.0**	N.A.	40.8–41.9/**24.6–27.9**	44.6/**27.6**	37.9/**24.2**	41.4/**30.3**	43.5–45.9/**20.3–23.4**		59.8/**64.1**	57.5/**61.2**
RdCmHEV	38.2–43.1/**17.8–27.1**	37.6–38.1/**17.7–22.6**	40.3–41.7/**23.3–25.0**	N.A.	40.5–41.3/**20.2–21.0**	44.5/**22.6**	39.6/**22.2**	43.3/**21.7**	40.2–42.3/**14.9–19.5**	34.2/**18.9**		58.5/**63.5**
RdCbHEV	36.6–39.4/**20.5–25.0**	36.9–38.0/**17.2–21.6**	37.6–37.9/**21.7–23.5**	N.A.	33.9–35.0/**16.0–20.2**	35.8/**21.8**	33.2/**15.6**	32.3/**19.7**	31.2–32.2/**15.9–21.4**	33.0/**15.9**	39.9/**18.9**	
	ORF3 (nt/aa)											

^1^ RdAcHEV, RdAaHEV, RdEmHEV, RdEiHEV, RdMrHEV, RdMgHEV, RdMaHEV, RdNlHEV, RdCmHEV, and RdCbHEV stand for HEV variants derived from the species *Apodemus chevrieri*, *Apodemus agrarius*, *Eothenomys melanogaster*, *Eothenomys inez*, *Myodes rufocanus*, *Microtus gregalis*, *Microtus arvalis*, *Necromys lasiurus*, *Cricetulus migratorius*, and *Cricetulus barabensis*, respectively. ^2^ Due to the sequence identity and phylogeny, two variants from humans (GenBank accession numbers MG813927 and MK050105) are included in the HEV-C1. ^3^ Nearly complete genome. ^4^ Due to the sequence identity and phylogeny, a variant from *Falco tinnunculus* (GenBank accession number KU670940) is included in the RdMaHEV. ^5^ N.A. stands for not available. Nucleotide (nt) and amino acid (aa) sequences were aligned using the MAFFT algorithm. Pairwise distances were computed in MEGA version 7. The GenBank Accession numbers are as follows: MH729810, MH729811, MH729812, MG813927, AB847307, JN167538, JX120573, JN167537, KM516906, MK050105, GU345043, AB847308, GU345042, AB847305, AB847306, AB847309, LC225388, LC225389, KY432898, and KY432906 for HEV-C1; AB890001, JN998606, JN998607, AB890374, LC057247, LC057248, LC177788, LC177789, LC177790, LC177791, and LC177792 for HEV-C2; MG020022 and MG020023 for RdAcHEV; KY432900 for RdAaHEV; KY432905, MG020024 and MG020025 for RdEmHEV; KY432904 for RdEiHEV; KY432901 for RdMrHEV; KY432902 for RdMgHEV; MK192405, MK192406, MK192407, MK192408, MK1924059, and KU670940 for RdMaHEV; MG021328 for RdNlHEV; KY432903 for RdCmHEV; and KY432899 for RdCbHEV. Lightface type indicates nt identity, and boldface type indicates aa identity. Viral strains from rodent families *Muridae* and *Cricetidae* and carnivore family *Mustelidae* are highlighted with grey, blue, and green background, respectively.
